# The prognostic value of immunoperoxidase staining with monoclonal antibodies NCRC-11 and 3E1.2 in breast cancer.

**DOI:** 10.1038/bjc.1991.254

**Published:** 1991-07

**Authors:** I. M. Muir, R. G. Reed, S. A. Stacker, A. I. Alexander, I. F. McKenzie, R. C. Bennett

**Affiliations:** Melbourne University Department of Surgery, St. Vincent's Hospital, Australia.

## Abstract

The variation in survival of women with clinically similar breast cancers may lead to difficulty in clinical management so it is important to recognise factors which indicate the prognosis. Immunoperoxidase staining patterns of primary breast tumours using monoclonal antibody NCRC-11 have been shown to relate to overall survival (Ellis et al., 1985) but the results have not been reproducible in other centres. In this study paraffin sections of 483 primary breast cancers were stained with NCRC-11 and 3E1.2 using an immunoperoxidase system. The tumour staining patterns were compared with overall survival using life tables and tested for relative prognostic significance by Cox's multivariate analysis. NCRC-11 related to survival in all 483 cases (chi 2 5.8, P = 0.02) but both antibodies achieved maximum prognostic significance in lymph node negative patients (chi 2 9.4, P less than 0.002 and chi 2 10.7, P less than 0.001) in whom no other factor was more significant. Immunoperoxidase staining patterns produced by monoclonal antibodies NCRC-11 and 3E1.2 are important prognostic factors in breast cancer.


Br. J. Cancer (1991), 64, 124-127 © Macmillan Press Ltd., 1991

### The prognostic value of immunoperoxidase staining with monoclonal antibodies NCRC-11 and 3E1.2 in breast cancer

I.M. Muir[1], R.G. Reed[2], S.A. Stacker[3], A.I. Alexander[1], I.F.C. McKenzie[3] & R.C. Bennett[1]

[1]*Melbourne University Department of Surgery, St. Vincent's Hospital;* [2]*Department of Pathology, St. Georges Hospital, Melbourne; and* [3]*Research Centre for Cancer and Transplantation, Department of Pathology, University of Melbourne, Australia.*

**Summary** The variation in survival of women with clinically similar breast cancers may lead to difficulty in clinical management so it is important to recognise factors which indicate the prognosis. Immunoperoxidase staining patterns of primary breast tumours using monoclonal antibody NCRC-11 have been shown to relate to overall survival (Ellis *et al.*, 1985) but the results have not been reproducible in other centres. In this study paraffin sections of 483 primary breast cancers were stained with NCRC-11 and 3E1.2 using an immunoperoxidase system. The tumour staining patterns were compared with overall survival using life tables and tested for relative prognostic significance by Cox's multivariate analysis. NCRC-11 related to survival in all 483 cases ($\chi^2$ 5.8, $P = 0.02$) but both antibodies achieved maximum prognostic significance in lymph node negative patients ($\chi^2$ 9.4, $P < 0.002$ and $\chi^2$ 10.7, $P < 0.001$) in whom no other factor was more significant. Immunoperoxidase staining patterns produced by monoclonal antibodies NCRC-11 and 3E1.2 are important prognostic factors in breast cancer.

The survival of women with clinically similar breast tumours may vary widely. In consequence the choice of treatment, the counselling of the patient and the interpretation of the results of clinical trials increasingly rely on laboratory tests of tumour biology in addition to clinical signs. Of all prognostic factors the histological diagnosis of lymph node metastases is the factor which relates most strongly to a poor prognosis and additionally the number and level of involved nodes affects survival (Fisher *et al.*, 1983). Factors relating to histological differentiation and hormone receptor status have also been found to have prognostic significance (Elston, 1984; Bryan *et al.*, 1986) but this in the former is observer dependent (Gilchrist & Kalish, 1985) and in the latter probably only applies in lymph node positive cases (Williams *et al.*, 1987). There is therefore a need to discover other prognostic factors which will help in patient management and in understanding more of the biology of breast cancer. This paper describes the investigation of the prognostic value of monoclonal antibodies (MoAbs) NCRC-11 and 3E1.2 in 483 women with primary breast cancer. Both of these MoAbs are of the anti-Epithelial Membrane Antigen (EMA) type and their characterisations and clinical uses have been described elsewhere (Ellis *et al.*, 1984, 1985; Stacker *et al.*, 1985, 1988, 1989). The synthesis of EMA by mammary acini and ducts is thought to be a specialised function and therefore tumours which contain EMA in abundance may be better differentiated and hence have a better prognosis than those in which it is less plentiful. In two studies using NCRC-11 and an immunoperoxidase method a relationship was demonstrated between well stained tumours and a favourable prognosis (Ellis *et al.*, 1985, 1987). In a smaller study this relationship could not be confirmed (Angus *et al.*, 1986) and no such study has been undertaken for 3E1.2. By using a large series of unselected patients followed-up for between 5 and 10 years, this study attempts to clarify the prognostic worth of both NCRC-11 and 3E1.2.

### Patients and methods

### *Clinico-pathological data*

Since 1976 the steroid receptor laboratory in the Department of Surgery at St Vincent's Hospital has assessed over 6,000 breast tumours. During this time it has been the policy of surgeons in the state of Victoria to submit specimens of all primary breast cancers for analysis. The patients for study were selected in sequence from the departmental records if the tumour was a primary breast cancer and the referring pathologist was prepared to release the appropriate paraffin block. No other selection criteria were applied. Four hundred and eighty-three patients were available for study with follow-up ranging from 5 to 10 years or to death. Treatment was not standardised though 87% received Patey mastectomy, 8% simple mastectomy and 5% lumpectomy. No data were available regarding treatment by radiotherapy or chemotherapy but in the time when these patients were treated there was no evidence to suggest that the parameters studied here would influence either a patient's selection for or response to this type of therapy. These patients represent a random sample of women with primary breast cancer treated in the state of Victoria between January 1976 and December 1981.

All patients had oestrogen receptor assays, 388 had progesterone receptor assays and 233 had androgen receptor assays. In 246 cases histological grade was assessed by one of us (RR) according to the criteria of Bloom and Richardson (1957) and in all cases histological type was known. Tumour size was known in 451 cases, and the number of involved nodes in 425 cases.

Follow-up information was obtained from the Commonwealth of Australia Electoral Register, the Anti-Cancer Council of Victoria and the referring surgeons and general practitioners.

### Technical methods

All archival material had been treated by formalin fixation alone with the exception of specimens from one laboratory which, in addition, used mercuric chloride secondary fixation. New paraffin sections were cut from the blocks, three were stained by a peroxidase anti-peroxidase (PAP) method (Ellis *et al.*, 1985) with NCRC-11, 3E1.2 and a negative control MoAb. A fourth section was stained by haematoxylin and eosin for assessment of histological grade. Prior to the present study an extensive assessment was made of other types of IP staining methods and the effect of many different regimes for tissue fixation. In breast cancer sections, with the MoAbs studied, the PAP method gave consistently reproducible results irrespective of fixation regime.

Assessment of MoAb stained sections consisted of light microscopic estimation of the percentage of carcinoma cells

Correspondence: I.M. Muir, Department of Surgery, Derbyshire Royal Infirmary, London Road, Derby DE1 2QY, UK.
Received 19 March 1990; and in revised form 11 February 1991.

stained irrespective of the part of the cell stained or the type of tumour. The percentage was then expressed as a score such that 0–25% scored 1, 26–50% scored 2, 51–75% scored 3 and tumours with greater than 75% of carcinoma cells staining scored 4. An average score is given when a tumour shows heterogeneity of staining. The scoring method has already been thoroughly documented (Muir *et al.*, 1987) and is not elaborated on here. It is easy to learn and has a high degree of observer reproducibility and interobserver correlation.

A computer file was compiled of the data for each patient and the file was analysed using the BMDP statistical software package (Dixon, 1983). MoAb staining was compared with other tumour data using chi-squared tests and Pearson's corelation coefficient then with survival using life table analysis. The prognostic value of staining score relative to that of other clinico-pathological data was assessed by Cox's multivariate analysis. Survival analyses were performed for all cases and separately for lymph node negative and lymph node positive cases.

### Results

Sixty-three per cent of tumours had identical staining scores with both MoAbs and in a further 27% the difference in score was only one unit. The scatterplot (Figure 1) shows that where staining scores were not equal, that NCRC-11 tends to stain more carcinoma cells then 3E1.2.

Staining score with both MoAbs was strongly related to oestrogen receptor status, progesterone receptor status and histological grade and weakly associated with age at the time of diagnosis (Table I). Staining did not relate to histological type, androgen receptor status, lymph node involvement or tumour size.

NCRC-11 staining score related to prognosis in all 483 cases, $\chi^2$ 5.8; $P<0.02$ (Figure 2) but the prognostic value is clearer when staining scores 1 and 2, 3 and 4, are amalgamated (Figure 3). This is consistent with the findings of Ellis *et al.* (1987).

In all 483 patients 3E1.2 staining did not have prognostic value though it approached statistical significance when staining scores 1 and 2, 3 and 4, were amalgamated, $\chi^2$ 3.4, $P=0.06$.

In the 223 lymph node negative patients both NCRC-11 and 3E1.2 staining scores related to survival (Figures 4, 5) with respective $\chi^2$ value 9.4, $P<0.002$, and 10.7, $P<0.001$.

In 202 lymph node positive patients staining score with neither of the MoAbs related to survival, $\chi^2$ 2.38 and 0.28. In this group the number of positive nodes ($\chi^2$ 5.5), tumour size ($\chi^2$ 7.5), oestrogen receptor status ($\chi^2$ 8.4) and progesterone receptor status ($\chi^2$ 10.4) related to survival.

The Cox Analysis for all 483 patients indicated that the following factors were associated with prognosis: lymph node status, number of involved nodes, tumour size, oestrogen receptor status, progesterone receptor status, age and NCRC-11 staining score. The factors found to have independent prognostic significance and a measure of their discrimination are shown in Table II. It is noteworthy that, once the prognostic value of progesterone receptor has been accounted for, oestrogen receptor status no longer had discriminatory power.

In node negative patients staining score with the 2 MoAbs

**Figure 1** Scatterplot showing comparative staining scores with 2 MoAbs in 483 cases.

**Figure 2** Life table showing survival according to NCRC-11 staining score in 483 cases.

**Table I** Statistical relationships between MoAb staining scores and other clinico-pathological data

| | *ER status* | *PR status* | *AR status* | *Histological grade* | *Age* | *Node status* | *Tumour size* |
|---|---|---|---|---|---|---|---|
| NCRC-11 | $\chi^2$ 34.3<br>3 d.f.<br>* | $\chi^2$ 22.9<br>3 d.f.<br>* | $\chi^2$ 3.8<br>3 d.f.<br>NS | $\chi^2$ 31.3<br>6 d.f.<br>* | R = 0.15<br><br>** | $\chi^2$ 7.1<br>3 d.f.<br>NS | R = 0.07<br>NS |
| 3E1.2 | $\chi^2$ 28.8<br>3 d.f.<br>* | $\chi^2$ 24.9<br>3 d.f.<br>* | $\chi^2$ 3.7<br>3 d.f.<br>NS | $\chi^2$ 27.4<br>6 d.f.<br>* | R = 0.14<br><br>** | $\chi^2$ 3.1<br>3 d.f.<br>NS | R = 0.07<br>NS |

*$P<0.0001$; **$P<0.001$; NS, not statistically significant; d.f., degrees of freedom; $\chi^2$ values were obtained from contingency tables, R = Pearson's correlation coefficient.

**Figure 3** Life table showing survival in 483 cases with MoAb NCRC-11. Staining scores 1 and 2, 3 and 4 have been combined.

**Figure 5** Life table showing survival in 223 lymph node negative cases with MoAb 3E1.2. Staining scores 1 and 2, 3 and 4 have been combined.

**Figure 4** Life table showing survival in 223 lymph node negative cases with MoAb NCRC-11. Staining scores 1 and 2, 3 and 4 have been combined.

**Table II** Order in which variables were removed from the Cox analysis for all 483 patients and the global chi-squared value for the total prognostic information

| *Variable* | *Chi-squared value* |
|---|---|
| Nodal status | 26.8 |
| PR status | 17.5 |
| Size (> or < 5 cms) | 16.5 |
| NCRC-11 | 5.8 |
| Nodes (1–3 or > 3) | 4.1 |
| Global chi-squared | 86.5 |

and progesterone receptor status related to survival. The Cox analysis showed that 3E1.2 staining score had the greatest discrimination in this group and neither of the other factors was sustained as having independent prognostic significance.

### Discussion

The results of this study confirm that NCRC-11 staining score is a powerful prognostic factor in patients with breast cancer. It is likely that the large number of patients studied and the long minimum follow-up have revealed a survival difference which has not come to light in smaller studies of NCRC-11 which have had shorter follow-up periods.

New discoveries are the relationship of 3E1.2 staining score to survival and, that for both MoAbs, the prognostic value is dependent on lymph node status. Both MoAbs had their maximum prognostic significance in lymph node negative patients but neither had prognostic value in node positive patients. It is therefore likely that the relationships demonstrated between NCRC-11, 3E1.2 and survival in all 483 cases are due to the influence of node negative patients rather than an ability of the MoAbs to discriminate between unselected cases. The effect of lymph node involvement on the discriminatory value of other prognostic factors has also been demonstrated for oestrogen receptor status (Williams, 1987) and epidermal growth factor status (Nicholson, 1989). In the present instance it is likely that the poor prognosis afforded by lymph node metastases outweighs any benefit from favourable MoAb staining. In node negative cases the degree of staining may relate to the predisposition of the tumour to form distant metastases or to the development of metastases in more or less lethal sites. In node negative cases MoAb staining score was the dominant prognostic factor. Progesterone receptor status was the only other factor significantly related to survival in this group but the Cox analysis showed that this was not independent of staining score.

MoAb staining score was not found to be a strong enough predictor of survival to discern more than two groups of patients, i.e. those with more or less than 50% of carcinoma cells stained. Of all the factors investigated only the extent of lymph node metastases possessed this property, i.e. groups 'O' nodes involved, '1–3' nodes involved and '4 or more' nodes involved. No relationship was demonstrated between histological grade and survival though favourable MoAb staining correlated with better histologically differentiated tumours.

It is concluded that immunoperoxidase staining score with NCRC-11 and 3E1.2 is a powerful prognostic factor in patients with breast cancer. This may have clinical significance in indicating which lymph node negative patients are at high risk and who may be considered for adjuvant treatment. It is suggested that investigators of other prognostic factors should stratify their results according to the lymph node status of their patients.

We are grateful to Dr R.A. Robins and Prof R.W. Baldwin of the Nottingham Cancer Research Campaign for kindly supplying NCRC-11 and to Drs J.P. Carew, N.A. Davies, A. Dorevitch, D.R. Hocking, M.P.K. Shoobridge and R. Zito for allowing access to archival tumour blocks from their laboratories.

### References

ANGUS, B., NAPIER, J., PURVIS, P. & 4 others (1986). Survival in breast cancer related to tumour oestrogen receptor status and immunohistochemical staining for NCRC-11. *J. Pathol.*, **149**, 301.

BLOOM, H.G.J. & RICHARDSON, W.W. (1957). Histological grading and prognosis in breast cancer. *Br. J. Cancer*, **11**, 359.

BRYAN, R.M., MERCER, R.J., BENNETT, R.C. & RENNIE, G.C. (1986). Prognostic factors in breast cancer and the development of a prognostic index. *Br. J. Surg.*, **73**, 267.

DIXON, W.J. (1983). *BMDP Statistical Software*. Berkely: UCLA Press.

ELLIS, I.O., ROBINS, R.A., ELSTON, C.W., BLAMEY, R.W., FERRY, B. & BALDWIN, R.W. (1984). A monoclonal antibody, NCRC-11, raised to human breast carcinoma. 1. production and immunohistological characterisation. *Histopathology*, **8**, 501.

ELLIS, I.O., HINTON, C.P., MCNAY, J. & 6 others (1985). Immunocytochemical staining of breast carcinoma with the monoclonal antibody NCRC-11: a new prognostic indicator. *Br. Med. J.*, **290**, 881.

ELLIS, I.O., BELL, J., TODD, J.H. & 5 others (1987). Evaluation of immunoreactivity with monoclonal antibody NCRC-11 in breast cancer. *Br. J. Cancer*, **56**, 295.

ELSTON, C.W. (1984). The assessment of histological grade in breast cancer. *Aust. N.Z.J. Surg.*, **54**, 11.

FISHER, B., BAUER, M., WICKERMAN, L., REDMOND, C.K. & FISHER, E.R. (1983). Relation of number of positive axillary nodes to the progress of patients with breast cancer. *Cancer*, **52**, 1551.

GILCHRIST, K.W. & KALISH, L. (1985). Interobserver reproducibility of histological features in stage II breast cancer. *Breast Cancer Res. Treat.*, **5**, 3.

MUIR, I.M., ELLIS, I.O., BELL, J. & ROBINS, R.A. (1987). NCRC-11 immunoperoxidase staining patterns in breast cancer: interpretive and technical reproducibility. *Histopathology*, **11**, 1208.

NICHOLSON, S., SAINSBURY, J.R.C., HARRIS, J.L. & FARNDON, J.R. (1989). Epidermal growth factor receptor status. A clinically useful prognostic indicator in axillary node negative breast cancer. *Abstract 15, Surgical Research Society of Great Britain and Ireland, Summer Meeting Newcastle-upon-Tyne*.

STACKER, S.A., THOMPSON, C.H., RIGLAR, C. & MCKENZIE, I.F.C. (1985). A new breast carcinoma antigen detected by a monoclonal antibody. *J. Natl Cancer Inst.*, **75**, 801.

STACKER, S.A., THOMPSON, C.H., SACKS, N.M.P. & 5 others (1988). Detection of mammary serum antigen from breast cancer patients using monoclonal antibody 3E1.2. *Cancer Res.*, **48**, 7060.

STACKER, S.A., TJANDRA, J.J., XING, P.-X. & MCKENZIE, I.F.C. (1989). Purification and biochemical characterisation of a novel breast carcinoma mucin-like glycoprotein defined by antibody 3E1.2. *Br. J. Cancer*, **59**, 544.

WILLIAMS, M.R., TODD, J.H., ELLIS, I.O. & 6 others (1987). Oestrogen receptors in primary and advanced breast cancer: an 8 year review of 704 cases. *Br. J. Cancer*, **55**, 67.


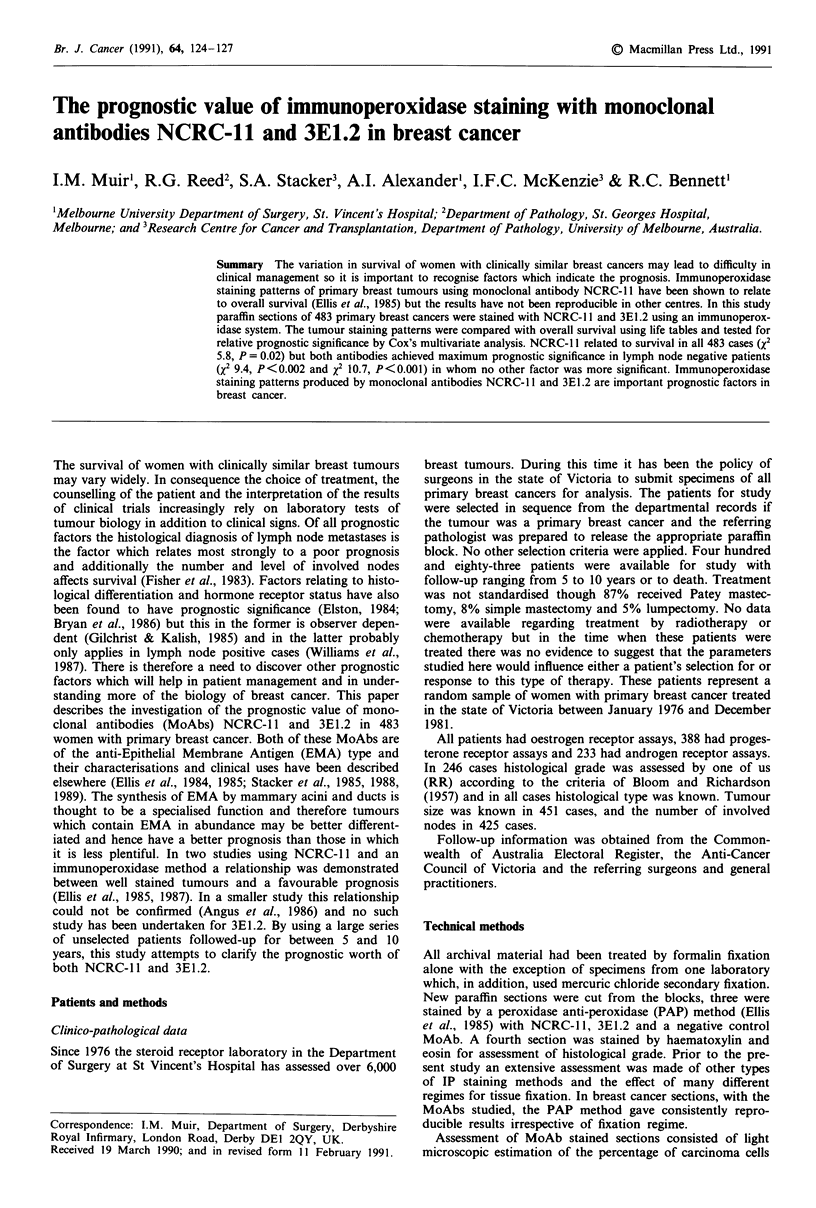

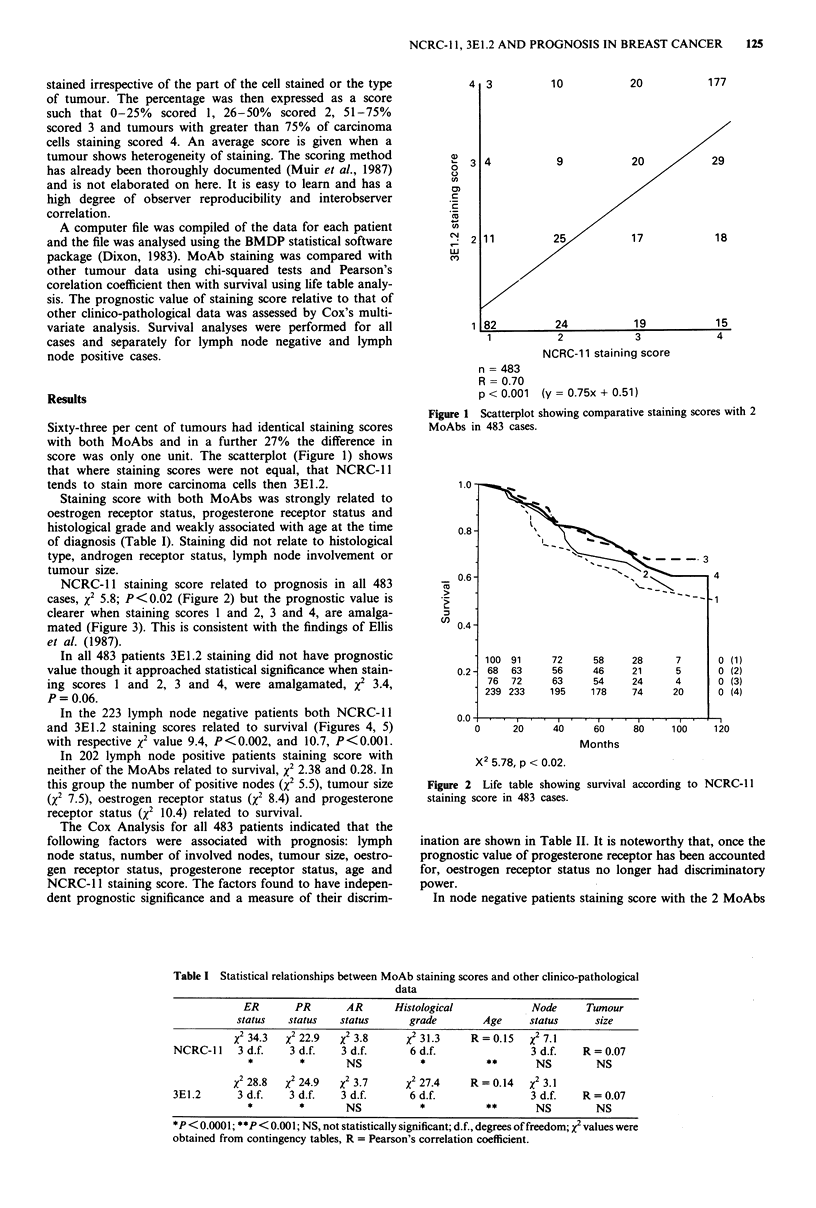

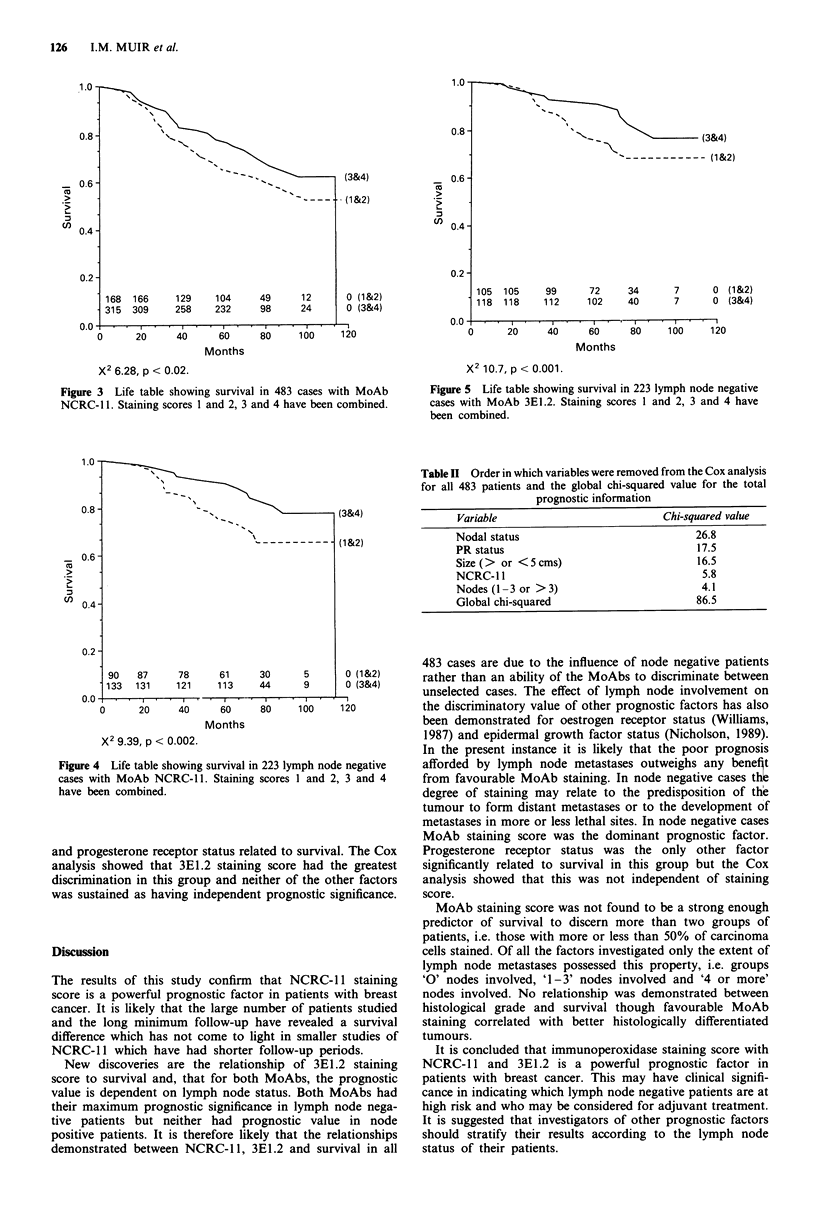

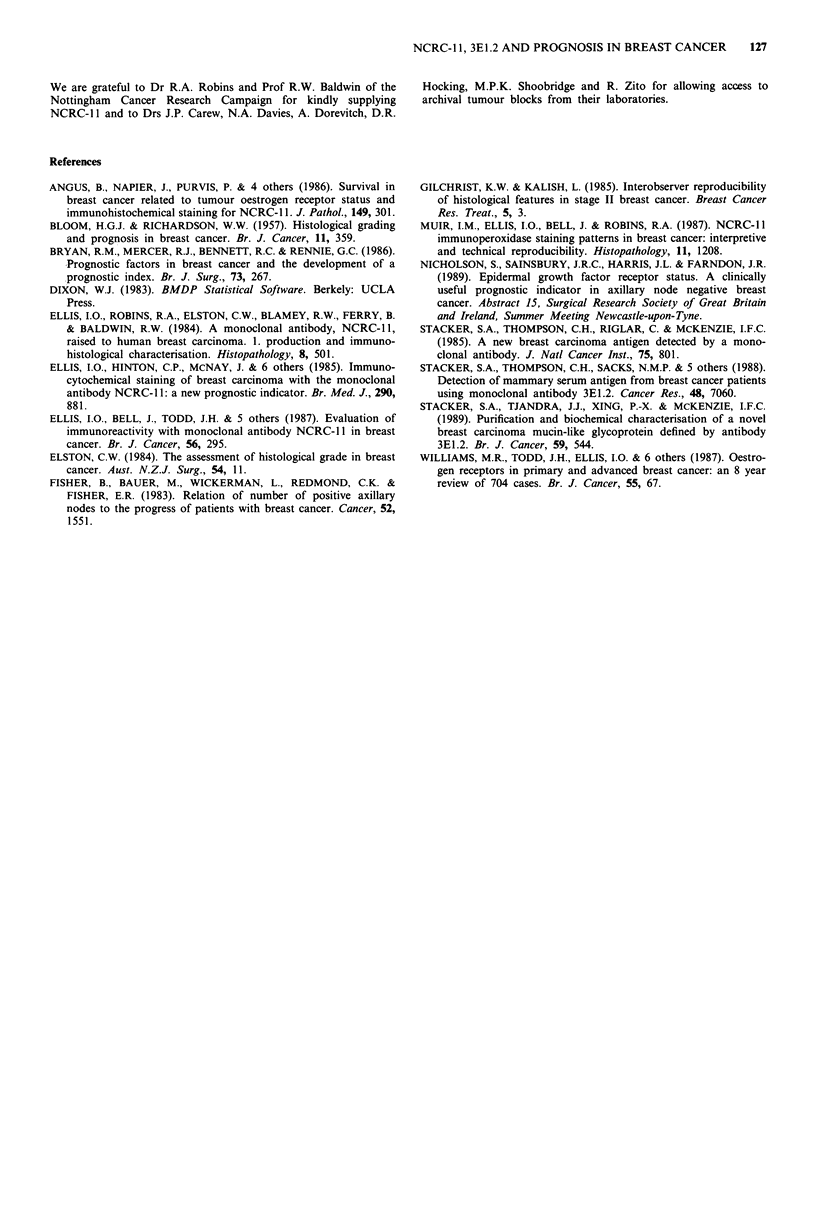


## References

[OCR_00440] Angus B., Napier J., Purvis J., Ellis I. O., Hawkins R. A., Carpenter F., Horne C. H. (1986). Survival in breast cancer related to tumour oestrogen receptor status and immunohistochemical staining for NCRC 11.. J Pathol.

[OCR_00444] BLOOM H. J., RICHARDSON W. W. (1957). Histological grading and prognosis in breast cancer; a study of 1409 cases of which 359 have been followed for 15 years.. Br J Cancer.

[OCR_00448] Bryan R. M., Mercer R. J., Bennett R. C., Rennie G. C. (1986). Prognostic factors in breast cancer and the development of a prognostic index.. Br J Surg.

[OCR_00469] Ellis I. O., Bell J., Todd J. M., Williams M., Dowle C., Robins A. R., Elston C. W., Blamey R. W., Baldwin R. W. (1987). Evaluation of immunoreactivity with monoclonal antibody NCRC 11 in breast carcinoma.. Br J Cancer.

[OCR_00463] Ellis I. O., Hinton C. P., MacNay J., Elston C. W., Robins A., Owainati A. A., Blamey R. W., Baldwin R. W., Ferry B. (1985). Immunocytochemical staining of breast carcinoma with the monoclonal antibody NCRC 11: a new prognostic indicator.. Br Med J (Clin Res Ed).

[OCR_00457] Ellis I. O., Robins R. A., Elston C. W., Blamey R. W., Ferry B., Baldwin R. W. (1984). A monoclonal antibody, NCRC-11, raised to human breast carcinoma. 1. Production and immunohistological characterization.. Histopathology.

[OCR_00478] Fisher B., Bauer M., Wickerham D. L., Redmond C. K., Fisher E. R., Cruz A. B., Foster R., Gardner B., Lerner H., Margolese R. (1983). Relation of number of positive axillary nodes to the prognosis of patients with primary breast cancer. An NSABP update.. Cancer.

[OCR_00484] Gilchrist K. W., Kalish L., Gould V. E., Hirschl S., Imbriglia J. E., Levy W. M., Patchefsky A. S., Penner D. W., Pickren J., Roth J. A. (1985). Interobserver reproducibility of histopathological features in stage II breast cancer. An ECOG study.. Breast Cancer Res Treat.

[OCR_00489] Muir I. M., Ellis I. O., Bell J., Robins R. A. (1987). NCRC-11 immunoperoxidase staining patterns in breast cancer: interpretive and technical reproducibility.. Histopathology.

[OCR_00506] Stacker S. A., Thompson C. H., Sacks N. P., Tjandra J., Lowe M. G., Bishop J., McKenzie I. F. (1988). Detection of mammary serum antigen in sera from breast cancer patients using monoclonal antibody 3E1.2.. Cancer Res.

[OCR_00501] Stacker S. A., Thompson C., Riglar C., McKenzie I. F. (1985). A new breast carcinoma antigen defined by a monoclonal antibody.. J Natl Cancer Inst.

[OCR_00511] Stacker S. A., Tjandra J. J., Xing P. X., Walker I. D., Thompson C. H., McKenzie I. F. (1989). Purification and biochemical characterisation of a novel breast carcinoma associated mucin-like glycoprotein defined by antibody 3E1.2.. Br J Cancer.

